# QuateXelero: An Accelerated Exact Network Motif Detection Algorithm

**DOI:** 10.1371/journal.pone.0068073

**Published:** 2013-07-18

**Authors:** Sahand Khakabimamaghani, Iman Sharafuddin, Norbert Dichter, Ina Koch, Ali Masoudi-Nejad

**Affiliations:** 1 Laboratory of Systems Biology and Bioinformatics, Institute of Biochemistry and Biophysics, University of Tehran, Tehran, Iran; 2 Molecular Bioinformatics, Johann Wolfgang Goethe-University, Frankfurt am Main, Germany; Universitat Rovira i Virgili, Spain

## Abstract

Finding motifs in biological, social, technological, and other types of networks has become a widespread method to gain more knowledge about these networks’ structure and function. However, this task is very computationally demanding, because it is highly associated with the graph isomorphism which is an NP problem (not known to belong to P or NP-complete subsets yet). Accordingly, this research is endeavoring to decrease the need to call NAUTY isomorphism detection method, which is the most time-consuming step in many existing algorithms. The work provides an extremely fast motif detection algorithm called QuateXelero, which has a Quaternary Tree data structure in the heart. The proposed algorithm is based on the well-known ESU (FANMOD) motif detection algorithm. The results of experiments on some standard model networks approve the overal superiority of the proposed algorithm, namely QuateXelero, compared with two of the fastest existing algorithms, G-Tries and Kavosh. QuateXelero is especially fastest in constructing the central data structure of the algorithm from scratch based on the input network.

## Introduction

Milo et al. [1] define “Network Motifs” as connectivity-patterns (subgraphs) in a particular network that occur much more often than they do in random networks. These patterns can be seen as the building blocks of networks. The importance of network motifs arises from the fact that they are closely related to many network properties such as structure, function, and robustness.

Since the introduction of this concept by Milo et al. in a seminal paper [1], a considerable number of researches have been conducted on this subject. Some of these researches focused on the biological aspects [2] [3] [4] and others concentrated on computational facets [5] [6] [7] [8] [9] [10]. The first group has endeavored to interpret the motifs detected in biological networks by the existing motif detection tools. But, the second group has tried to improve the existing motif detection tools to make this job easier for researchers of the first group. The current research belongs to the second group.

Motif detection in networks consists of two main steps: first, calculating the number of occurrences of a subgraph in the network and, second, evaluating the subgraph significance. Various methods proposed so far differ mainly in the first step, the enumeration of subgraphs. These methods can be grouped roughly into two categories regarding this aspect:

1. Methods counting subgraph occurrences exactly.
2. Methods using sampling and statistical approximations for the enumeration.

In this work, the focus is in the first category, which is also much more computationally demanding. The methods in this group require classifying the subgraphs after enumerating them in the network. In other words, the non-isomorphic classes of enumerated subgraphs should be determined. This can be done in two ways. First, one can generate all different non-isomorphic classes of a prescribed size and then calculate the frequency of each in the network (i.e., count the number of matches of each class in the network). The drawback is that the number of non-isomorphic classes grows exponentially with the given size of the subgraph. Grochow-Kellis [7] and MODA [11] exploits this approach. Second, one can perform the classification after the subgraphs are enumerated (i.e., for each enumerated subgraph we determine the non-isomorphic class separately). Faster tools, such as FANMOD [5], Kavosh [6] and G-Tries [8], use the latter classification method. This is also the approach used in the algorithm proposed in this paper.

The classification step is the most time consuming step of the second category methods. The reason is the application of isomorphism detection algorithms, mostly NAUTY [12], in this step. For example, in FANMOD and Kavosh, after enumerating each subgraph of a predefined size *s* it is first inputted to NAUTY algorithm, which produces a binary canonical labeling of length *s* ^2^ for that subgraph. Then, the canonical labeling is used as a key to search a binary tree, each leaf of which indicates a particular non-isomorphic class of size *s*. ESU, the algorithm used in FANMOD tool, is shown in Table 1: Algorithm 1 below (adapted from [13]).

**Table 1:** Algorithm 1.

*Algorithm 1:* ESU (FANMOD)
**Input:** Graph *G* and a positive integer *k*
**Output:** k-subgraphs census of graph *G*
1: **for all** *v*∈*V*(*G*) **do**
2: *V_Ext_*←{*u*∈*N*(*V*):*u*>*v*}
3: *EXTENDSUBGRAPH*(*V_Subg_*, *V_Ext_*, *v*, *QTree*.*root*)
4: **procedure** *EXTENDSUBGRAPH*(*V_Subg_*, *V_Ext_*, *v*, *CurQTNode*)
5: **if** |*V_Subg_*| = *k* **then**
6: *INCREMENTCOUNT*(*CANONICALLABELING*(*V_Subg_*))
7: **else**
8: **while** *V_Ext_*≠ø **do**
9: remove random chosen *w*∈*V_Ext_*
10: *V'_New_*←{*u*∈*N_Exclusive_*(*w*, *V_Subg_*): *u*>*v*}
11: *V'_Ext_*←*V_Ext_*∪*V'_New_*

The approach is different in G-Tries, in which a multi-way tree of depth *s*, the G-Trie, is used instead of the binary tree. However, again NAUTY is used for enumerating the subgraphs of the original network. But, the structure of the G-Trie tree is such that it can classify subgraphs of random networks without calling NAUTY. So, NAUTY is only used for census on the original network. This makes the G-Tries the fastest in the census on random networks.

Although NAUTY is one of the fastest isomorphism detection methods, but its computational cost is *O*(*s*!) in the worst case, which is very remarkable. Unfortunately, the isomorphism detection is an NP problem and no polynomial time algorithm is designed for solving it yet. Only a few methods, like SAUCY [14] and BLISS [15], have been designed for improving NAUTY’s performance in special cases, such as sparse graphs. However, still the upper bound is *O*(*s*!). Furthermore, searching the binary tree takes *s* ^2^−*s* operations, which is also considerable.

According to the above, it seems rational to search for methods that eliminate or decrease the number of executions of NAUTY in finding motifs. In fact, as stated above, this is the reason of G-Tries’s success as the fastest method so far. G-Tries algorithm eliminates the need to call NAUTY during the census on random networks. But, still, it uses the FANMOD for enumerating the subgraphs of the original network which is very time consuming and sometimes infeasible when the size of network and subgraph are large. G-Tries also provides other options that will improve its performance on original network, but applying these options need some prior knowledge or preprocessings. These options will be discuss later.

**Figure 1:**
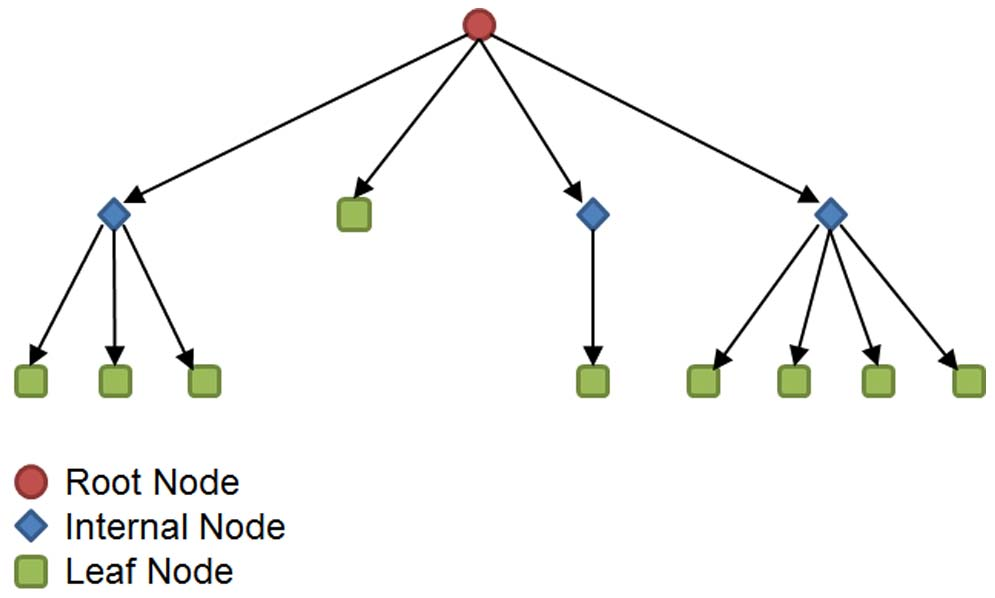
An example quaternary tree of depth 3. The root node and internal nodes have at most four children.

This paper provides a new algorithm with the aim of decreasing the number of calls to NAUTY. For this, the authors propose embedding a quaternary tree data structure in ESU (the algorithm used in FANMOD). A quaternary tree is a rooted tree data structure and each internal node has at most four children (see Figure 1). Accordingly, each internal node in the tree can have at most five neighbors, one of which is its parents and the others are its children.

**Figure 2:**
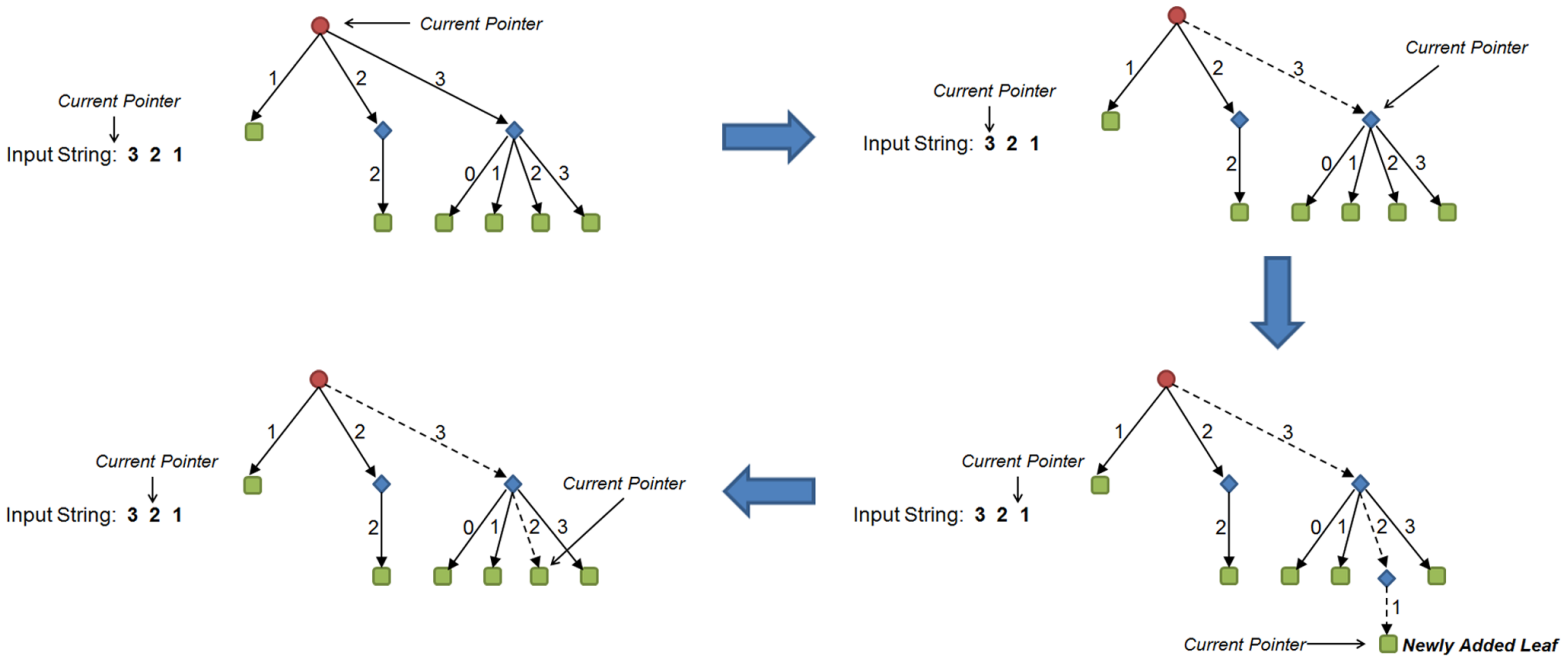
Searching a sample quaternary tree for input string “321”. Searching starts at the root of the tree. After respectively visiting children 3 and 2 throughout the path, the search finishes in a newly added leaf, corresponding to number 1.

Each edge, connecting a parent to one of its children, can be labeled with a mark, which can be a number, character, or any other symbol. A labeled quaternary tree can be searched using a given string that consists of the same set of symbols used for labeling that tree. This searching initiates in the tree’s root. In each step, one symbol is read from the input string and the current pointer, initially set as root, moves to the child of the current node, connecting edge of which corresponds to the symbol that is read recently from the input string. Because it is allowed to add nodes during the search, if one node in the path has no child for an input symbol, a child is added to the current node for that symbol and the current pointer moves to that child. Thus, this search continues until the input string is read completely. See Figure 2 for an example.

This quaternary tree performs a partial classification for enumerated subgraphs in the proposed algorithm. This data structure, which is similar to G-Trie data structure in some aspects, is used before calling NAUTY and eliminates the need to use it most of the times. According to experimental results, the proposed novel algorithm outperforms the existing algorithms in most of the cases.

## Materials and Methods

Like G-Tries, Kavosh, and FANMOD, QuateXelero consists of three main phases: enumeration, classification, and motif detection. Although enumeration and classification phases are intertwined, describing them separately makes them more understandable. Below, these phases are elaborated.

### Enumeration

For enumerating all subgraphs of size *k* in a given network, the general procedure is like the one in FANMOD algorithm. What makes the enumeration in QuateXelero different from that in FANMOD is the use of a quaternary tree. As in FANMOD the subgraph is extended by one vertex (hereafter, we use ‘vertex’ instead of ‘node’ when referring to the nodes of the input network, and alternatively, ‘node’ is used when referring to the nodes of the quaternary or binary trees) in each step, using the procedure *EXTENDSUBGRAPH*. However, this step by step extension allows the use of the quaternary tree, which is searched along with the extension. In other words, as the partial subgraph is extended by one vertex, the quaternary tree is also searched some levels further. Table 2: Algorithm 2 shows the algorithm of QuateXelero for census on the original network in detail.

**Table 2:** Algorithm 2.

*Algorithm 2:* QuateXelero (original network)
**Input:** Graph *G* and a positive integer *k*
**Output:** *k*-subgraphs census of graph *G*
1: **for** **all** *v*∈*V*(*G*) **do**
2: *V_Ext_*←{*u*∈*N*(*V*): *u*>*v*}
3: *EXTENDSUBGRAPH*(*V_Subg_*, *V_Ext_*, *v*, *QTree.root*)
4: **procedure** *EXTENDSUBGRAPH*(*V_Subg_*, *V_Ext_*, *v*, *CurQTNode*)
5: **if** |*V_Subg_*| = *k* **then**
6: **if** *CurQTNode.pointer* = NULL **then** *//Only in this case it is required to call NAUTY*
7: *CurQTNode.pointer*←*BLeaf*(*CANONICALLABELING*(*V_Subg_*)) *//BLeaf returns a pointer to corresponding leaf in the binary tree*
8: *INCREMENTCOUNT*(*CurQTNode.pointer*) *//Increases the counter of BLeaf to which the CurQTNode.pointer points*
9: **else**
10: **while** *V_Ext_*≠ø **do**
11: remove random chosen *w*∈*V_Ext_*
12: *CurQTNode'*←*SEARCH*(*V_Subg_*, *w*, *CurQTNode*) *//Searching the quaternary tree*
13: *V'_New_*←{*u*∈*N_Exclusive_*(*w*, *V_Subg_*): *u*>*v*}
14: *V'_Ext_*←*V_Ext_*∪*V'_New_*
15: *EXTENDSUBGRAPH*(*V_Subg_*∪{*w*}, *V'_Ext_*, *v*, *CurQTNode'*)
16: **procedure** *SEARCH*(*V_Subg_*, *w*, *CurNode*) **returns** *ResultNode*
17: *ResultNode*←*CurNode*
18: **for all** *u*∈*V_Subg_* **do**
19: **if** (*u*, *w*)∈*E*(*G*) **and** (*w*, *u*)∈*E*(*G*) **then**
20: *ResultNode*←child number 2 of *ResultNode*
21: **else if** (*w*, *u*)∈*E*(*G*) **then**
22: *ResultNode*←child number 1 of *ResultNode*
23: **else if** (*u*, *w*)∈*E*(*G*) **then**
24: *ResultNode*←child number −1 of *ResultNode*
25: **else**
26: *ResultNode*←child number 0 of *ResultNode*
27: **return** *ResultNode*

**Figure 3:**
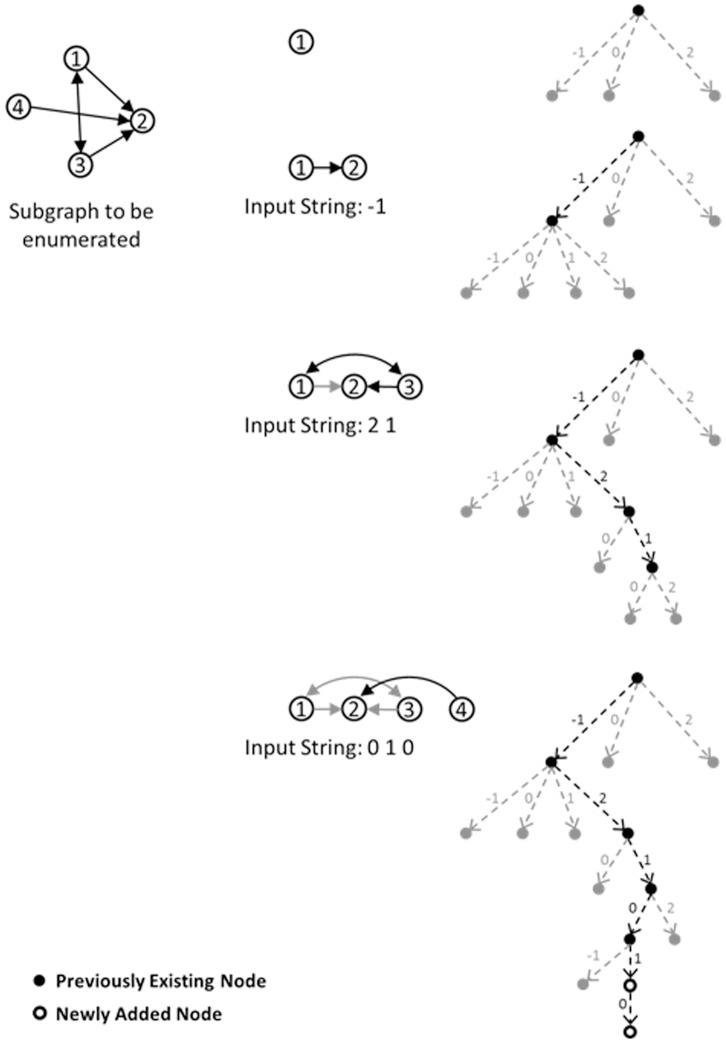
Steps taken to search the quaternary tree during expanding (enumerating) a sample subgraph. In this figure, −1 indicates one way connection from the existing vertex to added vertex, 0 indicates no connection between them, 1 stands for a one way connection in the reverse direction, and 2 shows a two way connection. The order of numbers in the input string is the same order as the corresponding vertices are added during expanding the subgraph (that is 1, 2, 3, and then 4 in this example).

Lines 6, 7, and 8 classify a subgraph after it is fully expanded. This is described in detail in the next section. Here, the *SEARCH* procedure is described. This procedure is called inside the function *EXTENDSUBGRAPH*, which expands the partial subgraph by one vertex each time it is called. After the new vertex w is selected from *V_Ext_* in line 11, the *SEARCH* procedure in line 12 uses the pattern of connections of *w* to other vertices of the partial subgraph (i.e. *V_Subg_*) to search the quaternary tree from *CurQTNode* to *CurQTNode'* which is |*V_Subg_*| nodes deeper (lines 17 to 27). It is notable that during this search the quaternary tree might be expanded with new nodes as described in section 2.1. The pattern of connections of *w* to other vertices of the partial subgraph is represented by a string of length *e* = |*V_Subg_*| consisting of the symbols {−1, 0, 1, 2} respectively indicating one way connection from a previously added vertex *u* in the subgraph to the newly added vertex *w*, no connection between these vertices, one way connection in the reverse direction, and a two way connection between them. An example of such a search is depicted in Figure 3. Since the procedure *EXTENDSUBGRAPH* is called *k*−1 times for a particular subgraph of size *k*, the total length of the path from the root of the quaternary tree to its leaf will be of length 1+2+ …+*k*−1 =  *k*(*k*−1)/2. This is the maximal complexity for procedure *SEARCH*. But, as a consequence of the recursive nature of the implementation, it is not needed to search the quaternary tree from the root for all subgraphs, so the complexity of the algorithm is reduced.

After searching the quaternary tree, the *V_Ext_* and *V_Subg_* sets are updated in lines 13 and 14 and the procedure *EXTENDSUBGRAPH* is recursively called based on these sets and the node *CurQTNode'*.

### Classification

**Figure 4:**
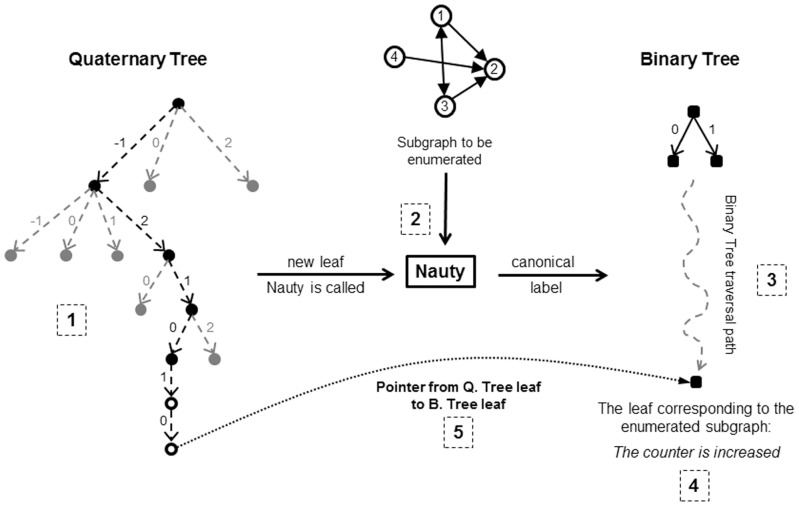
Steps taken during classifying a subgraph, in which a new leaf is added to the quaternary tree. 1) The quaternary tree is searched and the new leaf is added 2) Because the leaf is new and its pointers is not set, NAUTY is executed for the subgraph being enumerated 3) After finding the canonical label for the subgraph, the binary tree is searched using that label and the corresponding leaf in the binary tree is identified 4) The subgraph counter of that leaf (which indicates the number of subgraph of that class found so far in the network) is increase one unit 5) The pointer of the leaf of quaternary tree is set to the identified leaf of the Binary Tree.

**Figure 5:**
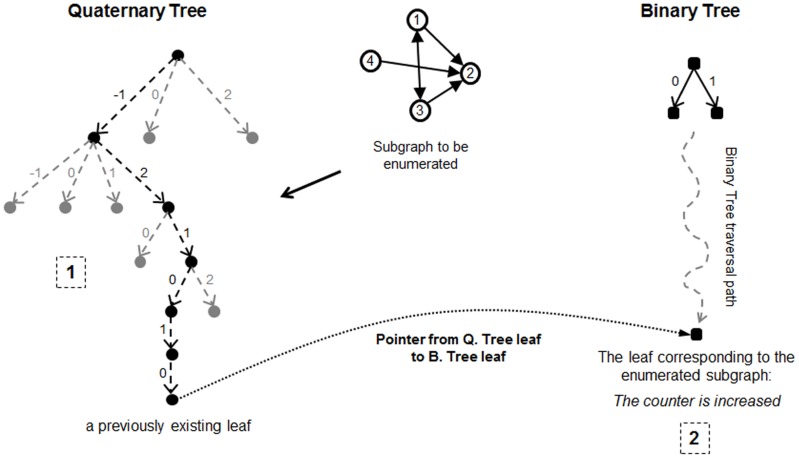
Steps taken during classifying a subgraph which has reached a previously existing leaf in the quaternary tree. 1) The quaternary tree is searched and the corresponding leaf is identified 2) Using the identified leaf’s pointer to the corresponding leaf from binary tree, the latter’s counter is augmented.

During the enumeration, the appropriate leaf of the quaternary tree is returned by the *SEARCH* procedure before the last call for *EXTENDSUBGRAPH* for a partial subgraph, in which the size of that subgraph reaches *k*. Then, the condition of ‘if’ in line 5 in Table 2: Algorithm 2 is satisfied. At this point, two cases might happen:

1. The *CurQTNode* is created during the search being performed for the current subgraph (see Figure 3): in this case, which is determined in line 6, it is needed to call NAUTY or *CANONICALLABELING* for the enumerated subgraph to determine its corresponding class which relates to a leaf in the binary tree. Then a pointer from *CurQTNode* is set to that leaf of the binary tree (see Figure 4). This is performed in line 7 of Table 2: Algorithm 2.
2. The leaf already existed in the tree and is not added newly: in this case, this leaf will have a previously set pointer to a leaf in the binary tree (i.e., the condition in line 6 is not satisfied) which indicates the isomorphism class to which the current subgraph belongs (see Figure 5). So there is no need to call NAUTY and search the binary tree for this subgraph.

In either of the above cases, the next step is to increase the counter of the corresponding leaf in the binary tree. This is performed in line 8 of Table 2: Algorithm 2, using the *CurQTNode.pointer* which points to the binary tree’s leaf.

The rationale underlying this classification is that if two different subgraphs reach the same leaf in the proposed quaternary tree, then those subgraphs are isomorphs of each other. But, it should be noted that the reverse is not true; in other words, it is possible for two isomorphic subgraphs to reach two different leafs of the quaternary tree. Thus, there may be two or more different quaternary tree leaves pointing to the same Binary Tree leaf.

Accordingly, in this algorithm (lines 6 to 7) the need to invoke the NAUTY function and searching the binary tree is eliminated in many cases by exploiting the proposed quaternary tree. That is, the cost of *s* ^2^−*s*+*O*(*s*!) is reduced to less than *s*(*s*−1)/2 for many of the enumerated subgraphs, while for others an extra *O*(*s*(*s*−1)/2) operation is added to *ss*+*O*(*s*!). But, how is the ratio of the former subgraphs (i.e., cost reduced) to the latter ones (i.e., cost augmented)? The answer to this question indicates the speedup ratio of the QuateXelero compared with Kavosh and FANMOD. As discussed in section 4, this highly depends on the number of non-isomorphic classes of the subgraphs of the given network. However, regarding the experimental results, in most cases, QuateXelero will perform remarkably better than existing algorithms, because the number of subgraphs is so much more than the number of non-isomorphic classes (especially in large biological networks). This means that a remarkable number of subgraphs will reach the same leaf of the quaternary tree, and so calling the NAUTY will not be required for them except for the first one. Consequently, this will significantly reduce the computational time of motif finding.

There is a delicate difference between census on the original network (Table 2: Algorithm 2) and the random networks in QuateXelero. During census on the original network, the binary tree would be modified when a new class of isomorphism is detected. However, for the random networks function *BLeaf* does not change the structure of a binary tree. It searches the binary tree until it reaches either a null node or a leaf. The former case means that the recently enumerated subgraph is of an isomorphism class that does not exist in the original network; so that the subgraph is ignored. In the latter case, the counter of the corresponding leaf in the binary tree is increased to account for the enumerated subgraph.

At the first glance, the algorithm might seem similar to the ESU option of G-Tries algorithm [8] (please refer to http://www.dcc.fc.up.pt/gtries/), but there are substantial differences. While the function of quaternary tree structure is the same as the G-Trie multi-way data structure and both have theoretically, but not practically, similar structures, it should be noted that the way of exploiting these data structures is completely different in two algorithms. First, like QuateXelero, the G-Tries structure is also constructed while processing the original network with the delicate difference that Quaternary Tree is developed along with enumerations but G-Trie is generated after the completion of enumerating the subgraphs of the original network (ESU). On the other hand, unlike QuateXelero, the canonical labeling is computed for all subgraphs of the original network in ESU step of G-Tries algorithm using NAUTY. This remarkably reduces the computational time of census on the original network in QuateXelero compared with G-Tries. Second, after constructing the G-Tries, NAUTY is not used any more for random networks, and instead the subgraphs are enumerated and classified using G-Tries data structure. But, in this work, the NAUTY is also possibly called for some subgraphs of random networks. However, this possibility gradually reduces during processing the random networks. Accordingly, it is the total number of executions of NAUTY in these algorithms that determines the superiority of one to another. Recall that NAUTY is the most time consuming part of the motif detection algorithms depending on it.

### Motif Detection

After the census on the original network with the help of a quaternary tree, each leaf of the binary tree will contain the number of subgraphs belonging to the corresponding isomorphism class. Then, some random networks are generated by rewiring and the census on is repeated on them. As the random generation method, we used the same method applied in G-Tries (3 swaps per edge with random Markov Chain process). The generated networks are checked against those generated by G-Tries and the results indicate the consistency of the random generation method.

Finally, the number of subgraphs of each isomorphism class for original and random networks will be used in calculating the z-score of each isomorphism class as below:
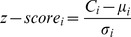
where *C_i_*, *µ_i_* and *σ_i_* are respectively the number of occurrences of *i* in the original network, average number of occurrences of *i* in the random networks, and the standard deviation of occurrences of *i* in the random networks. The higher the z-score, the more possible the particular isomorphism class (*i*) is a motif in the given network.

### Datasets

We used six standard networks for evaluating our algorithm. These were three biological networks: the metabolic pathway of bacteria *E. coli* [16], the transcription network of Yeast *S. cerevisiae* [17], and the protein-protein interaction network of the budding Yeast [18], [19], and three other non-biological networks: a real social network [6], a dolphins social network [20], [21] and an electronic network [1]. Self-loops were removed from all networks. The features of these networks are displayed in Table 3. All these datasets are included in the available online package for convenience.

**Table 3:** Experimental Datasets.

Network	Directionality	Vertices	Edges	Description	Source
Yeast	Directed	688	1079	Yeast transcription network	[17]
E. coli	Directed	672	1275	Metabolic pathway of bacteria *E. coli*	[16]
Social	Directed	67	182	A real social network	[6]
Electronic	Directed/Undirected	252	399 (both dir and undir)	Electronic circuit	[1]
YeastPPI	Undirected	2361	6646	Protein-protein interaction network in budding yeast	[19] and [18]
Dolphins	Undirected	62	159	Frequent associations between a group of dolphins	[21] and [20]

## Results

Because Kavosh and G-Tries are the bests amongst the existing motif finders, they are chosen for comparison with QuateXelero. G-Tries is superior regarding the speed and Kavosh is better in memory usage.

### Comparison with Kavosh

For comparing QuateXelero with Kavosh, both algorithms were executed on the same computer with Quad Core AMD Opteron ™ Processor 2354 and CentOS Linux Release 6.0 (final) operating system. The number of random networks is set to two in all experiments, which is enough for having valid results in experiments. It is important to note that this number of random networks is not suitable for motif detection in practice and is only used here for getting fast results for comparison. Moreover, different sizes of motif were considered in the experiments in order to assess the effect of the motif size on the performance of the algorithms.

The results are illustrated in Table 4. It is seen that, while QuateXelero is very faster than Kavosh in all cases, the amount of this superiority depends on the network size and structure, motif size, and the variety of its non-isomorphic classes. More precisely, it is completely related to the ratio of number of subgraphs to number of classes displayed in the fifth column of Table 4. The greater the ratio is, the more superior the performance of QuateXelero becomes. For example, QuateXelero is up to 86 times faster when finding motifs of size 8 in the Yeast network, but only 21 times faster for E.coli network in identifying motifs of size 9. This is mainly because the number of subgraphs in Yeast is greater than E.coli, but these subgraphs fall in a smaller number of non-isomorphic classes in Yeast compared with E.coli. So the need to call NAUTY is more reduced for Yeast than for E.coli.

**Table 4:** Experimental Results for QuateXelero vs. Kavosh.

					Processing Times		Comparison vs. Kavosh
Network	*s*	Subgraphs	Classes	Subgraphs/Classes	Kavosh	QX	
Yeast	5	2508149	174	14414.65	23.4	0.5	46.80x
Yeast	6	32883898	888	37031.42	438.5	8.9	49.27x
Yeast	7	416284878	4809	86563.71	14056.2	166.4	84.47x
Yeast	8	5184710063	27003	192004.97	224497	2609.5	86.03x
Yeast	9	64730339589	156025	414871.59	-	53852.1	-
Average Run Time Growth Ratio:					*22.3*	*18.2*	
Electronic	5	19675	49	401.53	0.13	0	nan
Electronic	6	97038	199	487.63	0.8	0.08	10.00x
Electronic	7	495274	907	546.06	5.9	0.3	19.67x
Electronic	8	2572125	4333	593.61	38.7	1.9	20.37x
Electronic	9	13512688	20692	653.04	278.2	11.9	23.38x
Electronic	10	71614362	96483	742.25	2614.2	71.2	36.72x
Electronic	11	381985209	437821	872.47	-	493.3	-
Average Run Time Growth Ratio:					*7.33*	*5.85*	
E.coli	5	80724	590	136.82	0.48	0.05	9.60x
E.coli	6	558080	3884	143.69	4.3	0.3	14.33x
E.coli	7	4019781	23587	170.42	45.3	2.8	16.18x
E.coli	8	29294103	136569	214.50	410.7	23.6	17.40x
E.coli	9	212782282	768121	277.02	4000	190.7	20.98x
Average Run Time Growth Ratio:					*9.57*	*7.96*	
Social	5	10599	773	13.71	0.11	0.06	1.83x
Social	6	52156	5062	10.30	0.82	0.36	2.28x
Social	7	254674	30217	8.43	5.4	2.6	2.08x
Social	8	1224376	165958	7.38	33.3	16.3	2.04x
Social	9	5764767	854023	6.75	220.3	96.22	2.29x
Average Run Time Growth Ratio:					*6.71*	*6.35*	

**Figure 6:**
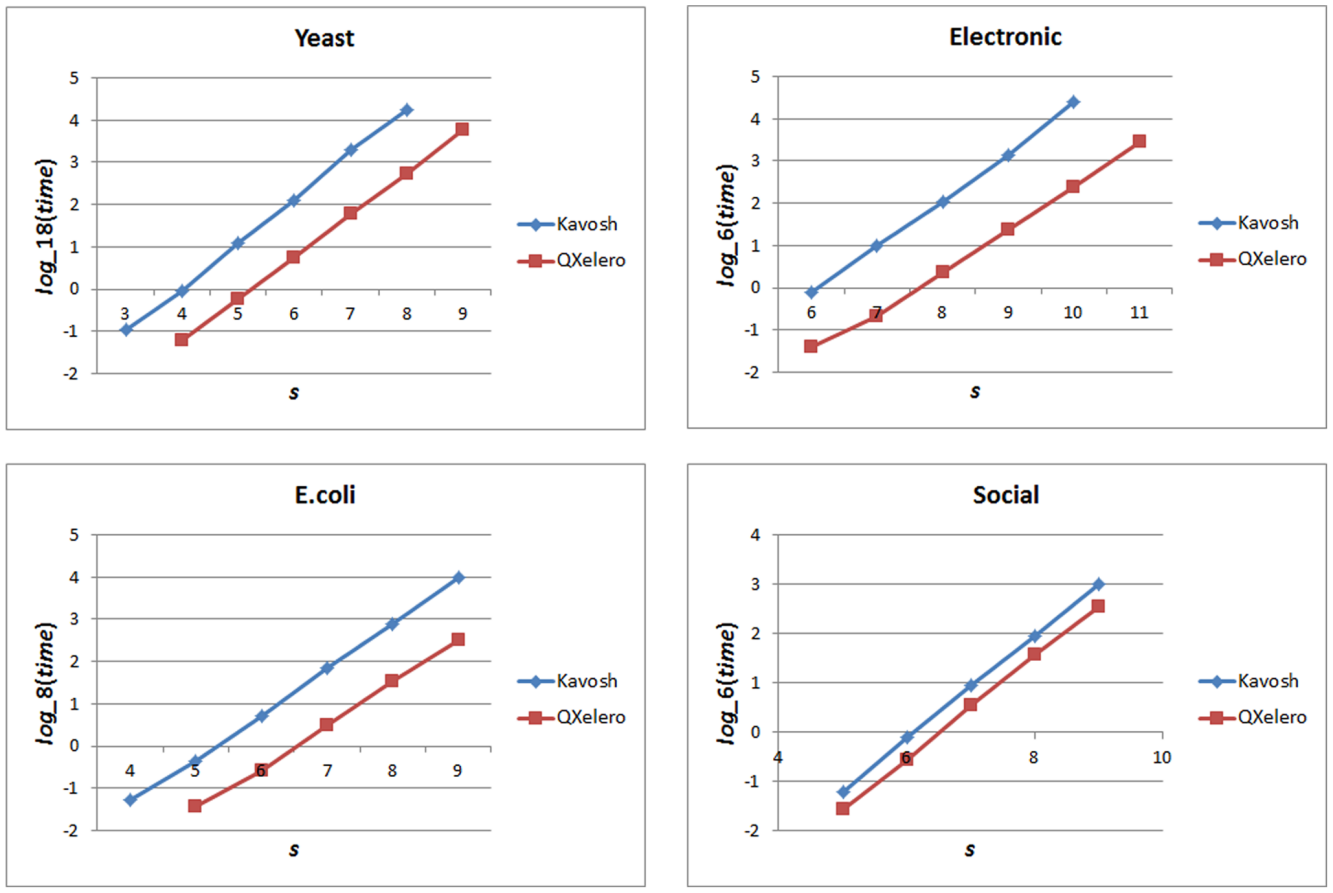
Growing gap between the running times of Kavosh and QuateXelero. In the charts, the horizontal axis indicates the size of motif and the vertical axis is the log of running time. The bases of logarithms are set to integer numbers close to the average running time growth rates shown in Table 5 for each network. The growing gaps are more visible in the charts for Yeast, Electronic, and E.coli networks.

However, generally, the results indicate that QuateXelero outperforms Kavosh regarding processing time in all cases. This is also illustrated in Figure 6, which also indicates the growing gap between algorithms when the size of the motif (i.e., *s*) is increased. In other words, QuateXelero still acts much better when the motif size increases. Average run time growth ratios in Table 4 further approve this fact.

The only drawback of the proposed algorithm is the considerable amount of memory that is used to construct the quaternary tree for larger motif sizes and for networks containing larger number of non-isomorphic subgraph classes. For example, among the experiments mentioned in Table 4, the highest amount of memory used by Kavosh was about 370 MB for Social network and motif size 9. On the other hand, QuateXelero occupied about 2.8 GB of memory (more than 7 times larger) for the same test case and about 4.6 GB for Electronic network and motif size 11. Nevertheless, regarding the availability and low prices of large memories nowadays, this could not be a very serious shortage, at least for smaller more popular sizes.

### Comparison with G-Tries

To compare QuateXelero with G-Tries, three groups of experiments are conducted. First, both of the algorithms are tested against smaller motif sizes on directed networks, second the same experiments are performed for larger sizes to understand the effects of motif size on run time of the two algorithms, and finally algorithms’ performances are tested for undirected networks.

Here, before explaining the experimental results, there is a point that worths noting. Currently, G-Tries provide an important and useful option for census on networks: having a list of non-isomorphic classes whose occurances are going to be counted, one can generate a G-Trie based on those subgraphs and then apply that G-Trie for enumerating subgraphs of both original and random networks.

However, it should be noted that if the goal is to exploit this option to enumerate all subgraphs occurring in a given network, two rough solutions might come to mind initially: 1) knowing all non-isomorphic classes occurring in the given network in advance, one can generate a G-Trie based on those subgraphs and then apply the G-Trie for enumeration, and 2) one can generate a G-Trie containing all possible non-isomorphic classes of a given size and then using it for enumeration. The first solution is obviously impossible as we need to first enumerate all subgraphs of a network before knowing their complete list of non-isomorphic classes. In other words, before being able to use this option to generate the solution, we need the solution itself. The second solution, although useful in smaller motif sizes, becomes impractical for sizes larger than 7 or 8 for directed and 11 or 12 for undirected networks, since the number of non-isomorphic classes grows exponentially and storing the generated G-Tries would need a tremendous amount of memory.

The provided option in G-Tries is useful when we are performing a set-centric subgraph enumeration (i.e., counting the occurances of a given set of subgraphs) or when the motif size is small. This option can (and is planned to) also be embedded in QuateXelero easily, as the general structure of QuateXelero and G-Tries are similar. However, the aim of this paper is not to compare the performance of two algorithms in set-centric searches, but this work is aimed at comparing these algorithms in both steps of generating and applying the Quaternary Tree and G-Trie data structures, specially for larger motifs where the set-centric option becomes inapplicable. Thus, here we emphasize the ESU option of G-Tries, which we call ESU+G-Tries. So the algorithm will have two steps: ESU (the algorithm of FANMOD) or census on original network, and G-Tries or census on randomized networks. The comparison of other options of G-Tries with the equivalent options in the proposed algorithm (which are planned to be implemented) takes a separate research.

Having said this, we continue discussing the comparison results. For comparing the algorithms a metric called “Equality Point” is defined. The equality point (*ep*) indicates the number of random networks, for which both algorithms take the same processing time to identify motifs. In other words, *ep* is the number of random networks at which the total processing times of both algorithms are equal. This can be calculated using the equation below, in which *t_o_^i^* is the time required by algorithm *i* for performing all calculations other than the census on random networks (including census on the original network, writing the output file, etc.), and *t_r_^i^* is the average time that an algorithm *i* spends for census on a single random network.
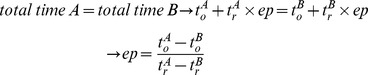



**Figure 7:**
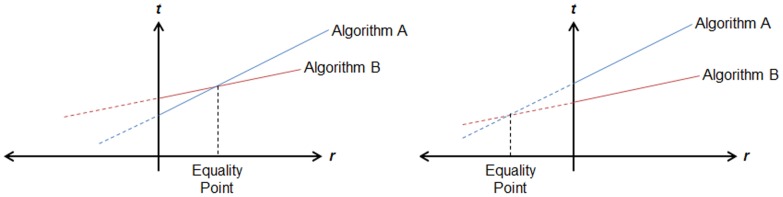
The concept of Equality Point. Positive and negative equality points are illustrated respectively in the left and the right charts. The vertical axis *t* indicates the total time of algorithms and the horizontal axis *r* shows the number of random networks used for motif detection.

This concept is also illustrated in Figure 7. This figure exhibits two different cases when the *ep* is positive (the left chart) and when it is negative (the right chart). In the former case, the equality point is the point after which the superior algorithm (i.e., A) becomes the inferior one, and the inferior one (i.e., B) becomes the superior. However, in the second case, one algorithm (e.g., Algorithm B) is superior to the other for all numbers of random networks. The *ep* metric is used later to investigate the usefulness of the proposed algorithm.

**Figure 8:**
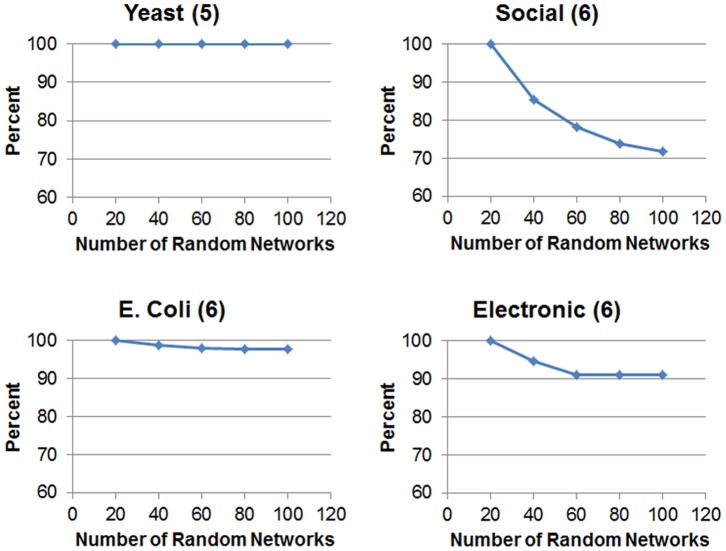
Effect of number of random networks on average time of census on a single random network. Numbers in the parenthesis show the size of the motif for which the experiments are conducted (the results can be generalized to other motif sizes). The vertical axis indicates the ratio (in percentage) of run time to the run time for 20 random networks. Except *Yeast*, the other networks exhibit a decline in the random network census time for the successive random networks.

First the results for the small motifs are discussed. These results are presented in Table 5. Before interpreting these results, there is a need to remark a significant feature of QuateXelero, which is not found in G-Tries. This feature is illustrated in Figure 8. This figure indicates that, except for Yeast, for all other networks the average time spend for census on random networks decreases as the number of random networks soars. This is especially observable for Social network, for which the variety of non-isomorphic classes is greater than for other networks. This phenomenon is the result of the fact that the quaternary tree becomes more and more complete when more random networks are enumerated using it. In other words, the more the variety of input subgraphs (i.e., more random networks), the more comprehensive the quaternary tree. So, the need to call NAUTY declines for the successive random networks and less time is spent on them. This fact was respected in designing the experiments for smaller motifs. Based on this phenomenon, the numbers of random networks for Yeast, Social, E.coli, and Electronic networks were set to 10, 100, 100, and 100, respectively. This was done with the assumption that many of the motif finding tasks uses 100 random networks in their calculations.

**Table 5:** QuateXelero (QX) vs. G-Tries in smaller motifs.

						Census on Original		Avg. Census on Randoms		Total Time		Memory		
Network	*s*	Subgraphs	Classes	Subgraphs/Classes	Avg. # of subgraphs in random nets	ESU of G-Tries	QX	G-Tries	QX	ESU+G-Tries	QX	G-Tries	QX	Equality Point
Yeast	5	2508149	174	14414.65	3277239	30.846	0.733	0.693	0.955	37.85	10.51	1.5 MB	1.8 MB	114.35
Yeast	6	32883898	888	37031.42	51982245	532.806	11.201	11.909	17.856	651.07	190.20	2.3 MB	2.5 MB	87.50
Yeast	7	416284878	4809	86563.71	872973082	12314.314	164.596	220.656	344.539	14494.60	3611.77	7.1 MB	8.8 MB	97.85
	*Average Growth Ratio:*			*2.45*	*16.33*	*20.19*	*14.99*	*17.86*	*19.00*					
Social	5	10559	773	13.66	16060.49	0.094	0.031	0.019	0.009	3.55	1.31	5.4 MB	2.7 MB	−124.00
Social	6	52156	5062	10.30	90430.94	0.581	0.218	0.118	0.070	22.74	9.00	30.7 MB	13.9 MB	−186.25
Social	7	254674	30217	8.43	499632.89	3.532	1.451	0.725	0.612	154.91	72.78	184.9 MB	143.7 MB	−626.81
	*Average Growth Ratio:*			*0.79*	*5.58*	*6.13*	*6.84*	*6.18*	*8.26*					
E.coli	5	80724	590	136.82	89831.69	0.612	0.063	0.126	0.037	15.63	5.51	4.5 MB	7.7 MB	−13.71
E.coli	6	558080	3884	143.69	639690.34	5.604	0.546	0.910	0.303	104.07	33.65	22.1 MB	13.6 MB	−16.01
E.coli	7	4019871	23587	170.43	4800418.40	51.092	4.430	7.195	2.600	822.42	274.45	135.4 MB	74.6 MB	−19.25
	*Average Growth Ratio:*			*1.12*	*7.30*	*9.14*	*8.39*	*7.56*	*8.39*					
Electronic	5	19675	49	401.53	20316.55	0.184	0.015	0.014	0.009	2.13	1.28	1.2 MB	3.4 MB	−70.00
Electronic	6	97038	199	487.63	99766.42	1.097	0.063	0.068	0.051	8.49	5.59	2.4 MB	3.9 MB	−70.59
Electronic	7	495274	907	546.06	522890.20	7.780	0.390	0.376	0.302	45.81	31.29	8.6 MB	8.5 MB	−96.22
	*Average Growth Ratio:*			*1.17*	*5.08*	*6.53*	*5.20*	*5.19*	*5.79*					

10, 100, 100, and 100 random networks were used respectively for Yeast, Social, E.coli, and Electronic networks.

Now, we return back to Table 5. It is seen in this table that in all cases, QuateXelero accomplishes census on the original network several times faster than ESU of G-Tries. However, on the other hand, G-Tries is faster in census on the random networks for Yeast. Again, with the assumption that most of the motif finding tasks uses 100 random networks and according to Equality Point values, it can be said that QuateXelero will detect motifs faster than ESU+G-Tries in all cases, except when finding motif of size 6 in the Yeast regulatory network, for which the *ep* is below 100. Both of the algorithms almost acts similarly for motifs of size 7 in the Yeast network (*ep* ≈ 100).

Taking into account the results for larger motifs shown in Table 6, it can be concluded that in Social and Electronic networks the performance of two algorithms converge as the size of motifs grows, and in a point, ESU+G-Tries would surpasses QuateXelero. For Social network, this has happened in Table 6, where the *ep* values are below 100. As stated in the previous section, this is partially related to the ratio subgraphs/classes displayed in column five, which is a very smaller value in Social network in comparison with other networks. Furthermore, unlike the other networks, for Social network this value decreases when the size of motif (i.e., s) is increased (i.e., its growth ratio is below 1). However, this is not the only factor influencing the Equality Point. Another factor is the degree distribution, which is closer to a normal distribution in Social network than the other networks, which have power-law distributions. Also, Social network has higher density (0.041) compared to Yeast (0.002), *E.coli* (0.003), and Electronic (0.006). All these factors augment the variety of subgraphs in random networks and so increase the possibility that QuateXelero calls NAUTY during the census on the random networks. This makes QuateXelero slower than ESU+G-Tries in detection of Social network’s large motifs when the number of the random networks is high. While QuateXelero has always been better in detecting the motifs of the Electronic network in our experiments, the trend of *ep* values indicates that ESU+G-Tries will surpass QuateXelero for larger motif sizes. These are also concludible according to the values of average growth ratios, as the average growth ratio of the time of census on random networks for QuateXelero (column 10) is always greater than the same value for G-Tries (column 9), except for large motifs of the *E.coli* network.

**Table 6:** QuateXelero (QX) vs. G-Tries in larger motifs.

						Census on Original		Avg. Census on Randoms		Total Time		Memory		
Network	*s*	Subgraphs	Classes	Subgraphs/Classes	Avg. # of subgraphs in random nets	ESU of G-Tries	QX	G-Tries	QX	ESU+G-Tries	QX	G-Tries	QX	Equality Point
Yeast	9	64730339589	156025	414871.6	255298149957	***848186.49***	6205.98	***13544.20***	23950.802	***915907.47***	125962.31	***711 M***	889 M	***80.91***
	*Average Growth Ratio:*			*-*	*-*	*-*	*-*	*-*	*-*					
Social	9	5764767	854023	6.8	15950595	21.25	9.62	5.830	12.228	119.34	83.37	1.5 G	2.8 G	10.62
Social	10	26429201	4161477	6.4	81106854	121.01	54.30	34.570	85.252	731.66	558.70	7.9 G	18 G	8.41
Social	11	117219394	19285152	6.1	392209489	669.35	273.54	237.761	653.014	4527.95	4368.38	40.0 G	59 G	5.38
	*Average Growth Ratio:*			*0.95*	*5.0*	*5.61*	*5.34*	*6.40*	*7.32*					
E.coli	9	212782828	768121	277.0	281406579	728.69	47.52	86.493	41.078	1223.27	264.96	1.2 G	2.4 G	−16.10
E.coli	10	1529707241	4223040	362.2	2564178587	6357.95	352.46	929.200	402.146	11461.69	2443.21	7.6 G	19 G	−12.11
E.coli	11	10854043472	22764206	476.8	13801545748	53819.37	-	8834.432	-	101184.86	-	44.0 G	-	
	*Average Growth Ratio:*			*1.31*	*7.3*	*8.60*	*7.42*	*10.13*	*9.79*					
Electronic	9	13512688	20692	653.0	17031795	65.89	2.34	2.360	2.604	79.18	16.11	42 M	130 M	263.48
Electronic	10	71614362	96483	742.3	78568259	483.41	13.89	11.626	14.962	550.76	90.76	206 M	678 M	142.89
Electronic	11	381985209	437821	872.5	464546660	3998.61	82.75	76.793	113.920	4438.76	663.11	1.0 G	4.6 G	106.70
Electronic	12	2045287405	1943681	1052.3	2450710026	-	504.40	-	796.268	-	4557.15	-	25 G	
	*Average Growth Ratio:*			*1.17*	*5.3*	*7.80*	*6.00*	*5.63*	*6.78*					

5 random networks were used in all experiments. Bolded italic values for Yeast network are estimated with respect to the results in Table 5.

For Yeast network the situation is different. While the limited experiments here are not enough to make a judgment about this, but regarding Tables 5 and 6, it can be inferred that *ep* values do not exhibit a meaningful trend for this network, and the two algorithms act almost equally with ESU+G-Tries, being somewhat superior in detecting larger motifs.

However, for *E.coli*, QuateXelero has always been superior to ESU+G-Tries, and the trend of *ep* values indicates that for larger motifs these values will remain negative, which shows that QuateXelero will also be better for those motif sizes.

**Figure 9:**
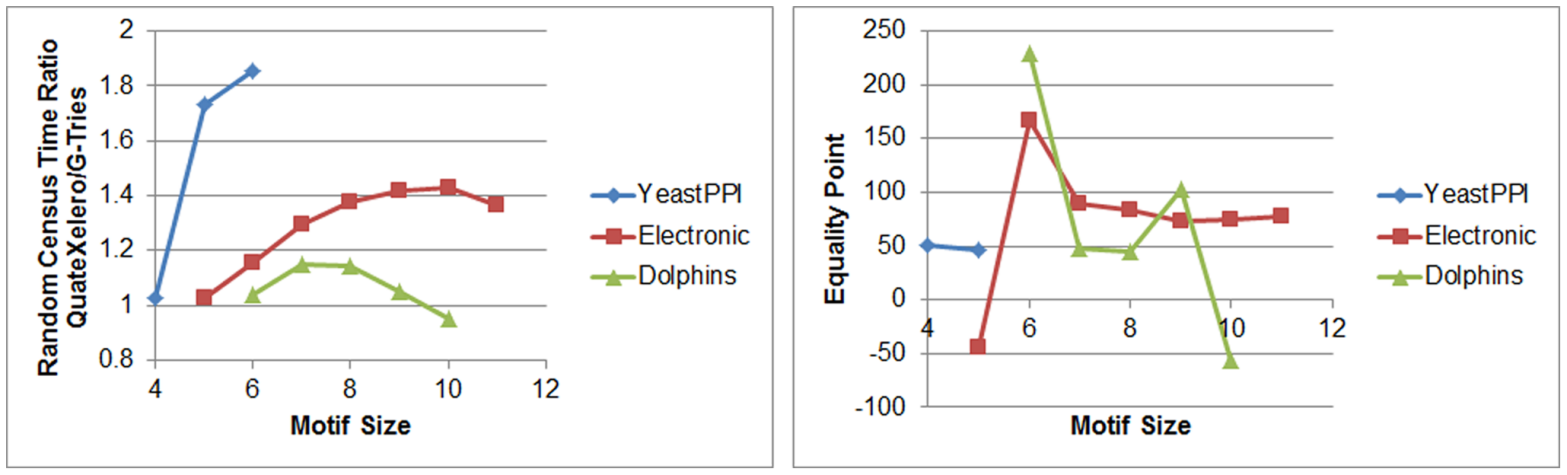
Trends of random network census time ratio (left) and Equality Point (right) for undirected networks. The ratio in the left chart indicates the ratio of average time spent by QuateXelero for census on random networks to the same time required for G-Tries.

The third series of experiments were about undirected networks. These results are displayed in Table 7 and Figure 9. From the table and figure, it can be understood that QuateXelero is faster for small and slower for medium size motifs. However, regarding the trends of random census time ratios (i.e. ratio of average time spent by QuateXelero for census on random networks to the same time required for G-Tries) and *ep* values, respectively in the left and right charts in Figure 9, it seems that the results for YeastPPI and Electronic will perform the same behavior observed for Dolphins in larger motif sizes. In other words, it seems that QuateXelero will again surpass in larger motifs, for which some limitations (time for YeastPPI and core dumping during running ESU+G-Tries for size 11 on Electronic network) prevented us from conducting more experiments. Furthermore, probabily there is a relationship between the ratio Subgraphs/Classes (column 4 of Table 7) and the performance of algorithms. Seemingly, QuateXelero will perform generally better for networks for which this ratio is small, as illustrated for Dolphins network.

**Table 7:** QuateXelero (QX) vs. G-Tries in undirected networks.

						Census on Original		Avg. Census on Randoms		Total Time		Memory		
Network	*s*	Subgraphs	Classes	Subgraphs/Classes	Avg. # of subgraphs in random nets	ESU of G-Tries	QX	G-Tries	QX	ESU+G-Tries	QX	G-Tries	QX	Equality Point
YeastPPI	4	2003998	6	333999.7	2447825	3.457	0.120	0.134	0.138	5.62	1.79	1.5 G	17.3 M	1031.26
YeastPPI	5	48870476	21	2327165.5	69599664	100.466	3.310	2.658	4.604	127.92	49.63	7.9 G	17.4 M	50.24
YeastPPI	6	1292780544	112	11542683.4	2158612083	3755.902	105.780	91.173	169.054	4667.06	1796.6	40.0 G	17.7 M	46.86
Electronic	5	19675	11	1788.6	21682	0.040	0.001	0.002	0.001	0.09	0.03	0.3 M	1.1 M	−43.90
Electronic	6	97038	33	2940.6	109648	0.267	0.010	0.010	0.012	0.39	0.14	0.4 M	1.1 M	166.26
Electronic	7	495274	89	5564.9	570167	1.544	0.060	0.056	0.073	2.13	0.81	0.6 M	1.5 M	89.25
Electronic	8	2572125	293	8778.6	3002254	10.118	0.370	0.310	0.427	13.26	4.65	1.3 M	3.0 M	83.26
Electronic	9	13512688	1001	13499.2	18291623	61.370	2.110	1.945	2.755	80.99	29.68	4.2 M	11.6 M	73.37
Electronic	10	71614360	3659	19572.1	104346200	393.620	12.110	11.872	16.959	513.26	181.82	19.3 M	49.7 M	75.15
Dolphins	6	107775	101	1067.1	251036	0.227	0.015	0.024	0.025	0.48	0.28	0.9 M	1.3 M	228.88
Dolphins	7	550428	633	869.6	1651879	1.338	0.140	0.174	0.200	3.18	2.20	1.8 M	2.6 M	47.88
Dolphins	8	2683740	4940	543.3	9602379	8.201	0.733	1.197	1.370	20.89	14.87	9.6 M	17.2 M	44.91
Dolphins	9	12495833	39963	312.7	53553629	42.379	4.618	8.403	8.800	133.24	96.27	79.8 M	170.2 M	103.15
Dolphins	10	55824707	295236	189.1	283463110	220.406	26.628	81.293	77.200	1096.96	828.15	638.3 M	1722.0 M	−55.68

10 random networks were used in all experiments. To save the time, we use less than 100 random networks here. This does not deteriorate the validity of results, because (1) undirected networks are not sensitive to the number of random networks as are the directed networks (please see figure 8) and (2) we do not base our analysis and comparison on the reported total time, but on the equality points and average random census times, which are independent of the total time.

Generally, regarding the experiments the followings can be concluded:

1. QuateXelero is always faster in census on original networks compared with ESU of G-Tries.
2. QuateXelero is generally faster in census on random networks for smaller motifs.
3. G-Tries is in most of the cases (especially for directed networks) faster in census on random networks for larger motif sizes.
4. QuateXelero is always better than ESU+G-Tries in the experienced motif sizes on E.coli network regardless of the number of random networks (negative *ep*) and probably would dominant in larger motif sizes too.
5. QuateXelero is generally better than ESU+G-Tries for smaller motif sizes.
6. QuateXelero surpasses ESU+G-Tries in most of our experiments for larger motif sizes in directed networks, however,it seems that ESU+G-Tries will be better for larger sizes not achievable with facilities available to the authors.
7. For undirected networks, QuateXelero surpasses ESU+G-Tries in smaller and seemingly larger motifs, however, ESU+G-Tries is better for medium size motifs.

There are two points that should be noted here. First, regarding the exponential growth in occupied memory, it seems infeasible to go further in motif size than what we have done, since it requires huge amounts of memory found only in limited scales in super-computers. Second, most of the current researches focus on motifs of size under 8, because the dynamical features of bigger motifs are yet unknown. Accordingly, the performed tests seem to be sufficient to provide reliable data.

For small size experiments, we employed a laptop computer with Intel Core™ 2 Duo CPU 2.5 GHz and 4 GB of RAM. For larger experiments, a master node of model Quad-Core AMD Opteron ™ Processor 2384 800 MHz with 64 GB main memory was used. The experiments for each network were conducted up to as large motif size as possible. However, some experiments were limited to the available memory and time. Generally, QuateXelero was mainly limited by the available memory while ESU+G-Tries was sometimes limited by time and sometimes by memory. These limitations and their details are listed in Table 8. Since the tests lasting more than 48 hours were cancelled, two first cases indicated in Table 8 were not completed. Accordingly, the results displayed in Table 6 for ESU+G-Tries in the case of finding motifs of size 9 in Yeast transcription network were estimated. The estimation was performed regarding results shown in Table 5. For this aim, the ratios of times used by QuateXelero for census on original and random networks to those times for ESU+G-Tries were traced regarding the values in Table 5. Then, we extrapolated these ratios for size 9 according to the trends recorded for sizes 5 to 7. Finally, by simply dividing the real times registered for QuateXelero by the extrapolated ratios, the estimated times for G-Tries were calculated.

**Table 8:** Experimental Limitations.

Network	Motif Size	Algorithm	Stopping Reason
Yeast	9	ESU+G-Tries	Long run time (close to 11 days)
Yeast	10	QX	Long run time (about 26 days)
Social	12	G-Tries	Memory
Social	12	QX	Memory
E.coli	12	G-Tries	Memory
E.coli	11	QX	Memory
Electronic	12	G-Tries	Core Dumped
Electronic	13	QX	Memory
Electronic (Undir)	11	G-Tries	Core Dumped

### Conclusions and Future Works

Network motif detection is a challenging problem regarding the computational time and memory it requires and there have been remarkable efforts to solve it efficiently. This paper provides a new solution for this problem which is claimed to be superior in terms of processing time to the existing solutions in special cases. This claim is approved with respect to the experimental results on some standard complex networks. The results of comparing the proposed algorithm, namely QuateXelero, with the well-known existing method Kavosh indicated the superiority of it to Kavosh in all cases regarding processing time. But QuateXelero uses a massive amount of memory compared with Kavosh. Another more important analysis was the comparison against ESU+G-Tries algorithm (ESU option of G-Tries algorithm). Generally, the results indicate that QuateXelero is always much faster than ESU of G-Tries in constructing the central data structure (i.e., the census on the original network), but slower in the census on random networks for larger motif sizes in most of the directed cases. The results for undirected networks illustrate the superiority of QuateXelero in small and probabily large motif detection, but not in the medium size problems. Furthermore, while QuateXelero is faster in most of the attempted experiences, but it seems that two algorithms, QuateXelero and ESU+G-Tries, will converge and the situation will be reverse when the size of the *directed* motif is set to numbers greater than those tested here. However, it should be noted that greater motifs are only detectable by using huge main memories, which might be only found in special super-computers. Moreover, current research does not exhibit a tendency towards larger motifs that what we have discussed.

Anyway, the proposed algorithm still seems to be improvable. With respect to the above, the future works can be focused on comparing the other options of G-Tries algorithm with the equivalent options in QuateXelero. Besides, combining the strength points of QuateXelero (e.g., faster census on original network) with the strength points of G-Tries (e.g., generally faster census on random networks and less memory occupation), to achieve a more efficient motif detection tool for solving problems in which the motif size is large and so other options are infeasible is another topic for further reseach. Furthermore, the question “When is QuateXelero faster than G-Tries or vice versa in the census on random networks?” is not answered completely yet. So, another point of focus can be the development of a strategy for choosing the appropriate method between two algorithms for census on random networks in processing a particular input network. Finally, one can use more compact data structures to compress the size of constructed quaternary tree to improve the memory complexity of QuateXelero.

### Implementation and Availability

QuateXelero is implemented in C++ programming language under Linux operating system. The program is also applicable under Windows (please refer to help file). The source code and sample networks are available for download at: http://lbb.ut.ac.ir/Download/LBBsoft/QuateXelero/.
